# A randomised, prospective study of the effects of 3% diquafosol on ocular surface following cataract surgery

**DOI:** 10.1038/s41598-021-88589-7

**Published:** 2021-04-27

**Authors:** Sangyoon Kim, Jonghoon Shin, Ji Eun Lee

**Affiliations:** 1 Department of Ophthalmology, Pusan National University School of Medicine, Yangsan, South Korea; 2 Department of Ophthalmology, Research Institute for Convergence of Biomedical Science and Technology, Pusan National University Yangsan Hospital, Yangsan, South Korea

**Keywords:** Randomized controlled trials, Preventive medicine

## Abstract

There is still no established therapeutic solution for postoperative Dry Eye Syndrome (DES) after cataract surgery, in spite of progress in surgical techniques. Diquafosol tetrasodium (DQS), a recently developed ophthalmic solution, has been reported to be effective in DES, but no study evaluated post-cataract surgery lipid layer thickness (LLT) changes in healthy patients who used DQS postoperatively. We randomly divided participants into two groups; the DQS group was treated six times daily with DQS after cataract surgery, and the sodium hyaluronate (HA) group was treated with HA in the same way. Throughout study period, the DQS group showed significantly higher tear break up time (TBUT) and LLT than HA group. In multivariate analysis, better preoperative TBUT, Schirmer’s I test score, ocular surface disease index (OSDI) score, and LLT were significantly associated with improved postoperative outcomes in each parameter. Also, the postoperative use of DQS served as an independent parameter of better TBUT, OSDI score, and LLT in postoperative 15 weeks. Treatment with 3% DQS following cataract surgery showed more improvement in TBUT and LLT, compared with 0.1% HA. Improving TBUT and LLT preoperatively and using 3% DQS postoperatively, could be a reliable choice for managing DES after cataract surgery.

**Trial Registration:** ISRCTN registry with ISRCTN 18755487.

## Introduction

Cataract surgery is one of the oldest and most common procedures performed worldwide. For centuries, improvements in surgical techniques have been made, and now it is commonly accepted as both safe and effective surgery in developed world. However, despite advancements in surgical techniques, many patients still struggle with postoperative eye symptoms, generally termed as ‘Dry eye syndrome (DES)’, such as ocular soreness, foreign-body sensation, and visual disturbance^1–3^. DES decreases the visual function in patients and reduce their ability to perform daily visual tasks. This, in turn, reduces their quality of life. The prevalence of these symptoms after cataract surgery was reported to range from 9.8 to 55.7%^1,2,4^. Accordingly, researchers have tried many eye solutions such as sodium hyaluronate (HA) to treat DES, many of which have been reported to be effective. However, variabilities in the cohorts, types of agents used, and follow-up periods among the studies pose difficulties in determining the best medication or treatment protocol for postoperative dry eye^5–8^.

Diquafosol tetrasodium (DQS) has been introduced as a topical medication for DES. It is a purinergic receptor agonist that binds to specific receptors on ocular surface and stimulates the secretion of aqueous tears and mucin, thereby stabilising the tear film. Several studies reported its effectiveness in reducing DES symptoms^1,9,10^. However, only a few studies demonstrated the effect of DQS on dry eye symptoms after cataract surgery. In addition, an objective postoperative evaluation of the tear lipid layer using an image-based diagnostic tool, such as the LipiView interferometer, in healthy patients has not been conducted.

The aim of this study was to compare the efficacy of a 3% DQS with that of a 0.1% HA eye solution on ocular surface after cataract surgery in healthy eyes by quantitatively evaluating clinical DES indices such as tear break up time (TBUT), Schirmer’s I test score, ocular surface disease index (OSDI) score, and lipid layer thickness (LLT).

## Materials and methods

The CONSORT checklist and protocol for this clinical trial is available in related files. This was a prospective, randomised, double-masked, and controlled clinical trial evaluating the efficacy of 3% DQS in terms of TBUT, Schirmer's I test score, OSDI score, and LLT in subjects after cataract surgery. The study protocol was approved by the Pusan National University Yangsan Hospital Institutional Review Board (No. 05-2019-200, 20/02/2020), and the study was performed in accordance with the tenets of the Declaration of Helsinki. The subjects were provided informed consent for participation in the study. The recruitment start date for the study was 20 February 2020, and the recruitment end date was 18 July 2020. The study was conducted between 20 February 2020 and 09 November 2020, at Pusan National University Yansan Hospital, Yangsan, Korea, and was registered with the International Standard Randomized Controlled Trial Number (Registration number : ISRCTN 18755487).

### Subjects

We followed the same protocol of the previous study in recruiting and enrolling the subjects^11^. The subjects included in the study were adults with cataracts who exhibited normal lid position and closure and did not have any ocular diseases. We excluded subjects who had used topical artificial tears, anti-inflammatory agents, antibiotics, or other medications, that could dry out the eye or stimulate tear secretion, during 90 days before the surgery. Patients with a history of eye trauma, ocular surgery, laser or systemic treatment known to affect tear secretion, autoimmune diseases, or slit-lamp evidence of eye surface disorders or those using contact lenses were also excluded. In addition, to exclude subjects with dry eyes from the study, the subjects were preoperatively required to show a normal fluorescent TBUT (> 10 s) and Schirmer’s I test score (> 10 mm with anaesthesia).

In total, 60 participants (60 eyes) were recruited, of which 56 (56 eyes) were enrolled in the study at the Department of Ophthalmology of Pusan National University Yangsan Hospital. Four patients were excluded because they had undergone previous treatments for ocular surface diseases. The sample size was calculated using MedCalc version 10.0 (MedCalc, Ostend, Mariakerke, Belgium). The minimum sample size requirement for a *t*-test with an alpha level of 0.05, and a power of 0.8, was calculated to be 21 for each group. Considering a 25% dropout rate, 28 participants were enrolled for each group. Four subjects in the HA group were excluded, as they were lost to follow-up. Eligible subjects were enrolled in the study and assigned a sequential number with a corresponding randomisation code generated by an independent third party using the SAS version 8.0 (SAS Institute, Inc., Cary, NC).

According to the randomisation protocol, the clinical staff assigned the subjects to receive either 3% DQS ophthalmic solution (Diquas-S; Mitsubishi Tanabe Pharma, Inc., Osaka, Japan) or 0.1% HA (HyalQ; Ildong Pharmaceutical, Inc., Seoul, Korea) six times daily for 15 weeks after cataract surgery. The clinical staff provided instructions on how to administer ophthalmic solutions. To achieve blinding of the researchers and subjects, the medications were filled in vehicles by a pharmacologist, and the type of each topical medication was not revealed until the completion of follow-up examination at the end of the study.

All subjects underwent standard small-incision cataract surgery performed by a single surgeon (JEL). A clear corneal incision 2.8 mm in length was made at the superotemporal region of the eye. All eyes received identical postoperative eye drops with a combination of 1.5% levofloxacin four times daily for 2 weeks and 0.1% fluorometholone four times daily for 1 week, followed by weekly tapering doses and either 3% DQS or 0.1% HA six times daily for 15 weeks.

### Clinical measurements

To assess the ocular surface status, the following measurements were preoperatively and postoperatively performed: 1-week preoperative TBUT, Schirmer’s I test score, and LLT. After the cataract surgery, the follow-up visits took place at 3, 7, and 15 weeks to measure postoperative TBUT, Schirmer’s I test score, and LLT at every visit. The ocular symptoms were evaluated using the OSDI score questionnaire at preoperative and every follow-up visit. The LLT was measured using the LipiView Ocular Surface Interferometer (TearScience Inc, Morrisville, NC) to obtain the interferometric image of the tear film as described previously^11^. Briefly, Interferometric colour units (ICUs) were used to measure LLT by the interferometer, with 1 ICU equal to 1 nm of LLT. The following measurements were recorded for each subject: average LLT obtained from all frame averages and the maximum and minimum LLT. Subjects with interferometer results showing a C-factor of less than 0.8 were excluded from the present study. LipiView had an upper cut-off of 100 ICU. The primary outcomes were to evaluate the changes in TBUT, Schirmer’s I test score, OSDI score, and LLT during the follow-up period between the DQS group and the HA group. The secondary outcomes were to determine the baseline factors that affected each clinical parameter at postoperative 15 weeks. To control subjective measure bias, the TBUT and OSDI score measurements were conducted by the same surgeon (JEL) across different time points.

### Statistical analysis

All statistical analyses were performed using SPSS for Windows version 26.0 (SPSS Inc., Chicago, IL). Descriptive statistics are presented as mean ± standard deviation. Data normality was verified using the Kolmogorov–Smirnov test. An independent *t*-test or chi-square analysis was used to compare the baseline values between the DQS study and HA groups. The time course of statistical changes in the values of TBUT, Schirmer’s I test score, OSDI score, and LLT between the two groups was evaluated by repeated measures analysis of variance (ANOVA). To compare the parameters for time points in each group, ANOVA, with post-hoc paired Tukey’s test was performed. Multiple linear regression analysis was used to identify the determinant factors associated with the clinical parameters, TBUT, Schirmer’s I test score, OSDI score, and LLT postoperatively at 15 weeks. Each variable was initially analysed using a univariate model; all significant variables (*p* < 0.10) were subsequently evaluated by a multivariate model using the backward method. The coefficient of determination (R^2^) in the linear regression was reported, and *p* < 0.05 was considered statistically significant.

### Ethics approval

Written informed consents were obtained from all participants. The study protocol and informed consent were approved by the Institutional Review Board at the Pusan National University Yangsan Hospital and all research was conducted in accordance with the Declaration of Helsinki.

## Results

**Figure 1:**
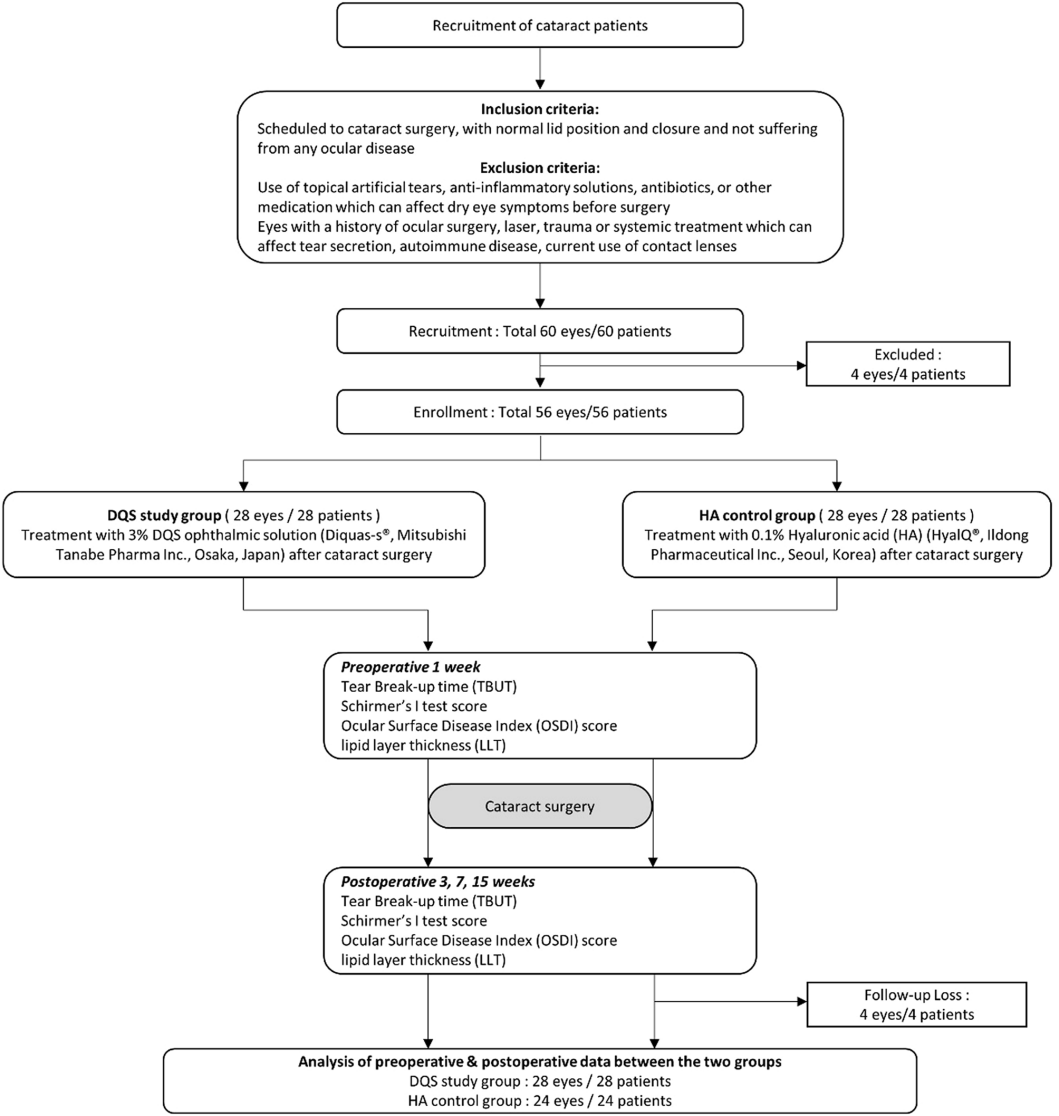
CONSORT protocol for the study described with flowchart.

Fifty-six subjects (56 eyes) were enrolled and randomised to the DQS and HA groups in a 1:1 ratio. Four subjects (4 eyes) from the HA group were excluded, as they were lost to follow-up within 4 weeks after cataract surgery. In this per-protocol dataset comprising 52 patients (52 eyes), 28 subjects (11 males and 17 females) treated using 3% DQS in the study group and 24 subjects (11 males and 13 females) treated with HA in the group were analysed (Fig. 1).

Results were reported for patients who completed the study and for whom all data were obtained for postoperative 3, 7, and 15 weeks. The clinical and demographic data of both groups are detailed in Table 1. At the preoperative visit, the mean TBUT, Schirmer’s I test score, OSDI score, and LLT were not significantly different between the DQS and HA groups (*p* = 0.177, 0.331, 0.287, and 0.065, respectively).

**Table 1:** Preoperative clinical characteristics and demographics.

Parameter	DQS group	HA group	*p* value
Age (year)	69.57 ± 6.22	67.08 ± 7.73	0.205^a^
Gender (male/female)	11/17	11/13	0.227^b^
Laterality (right/left)	18/10	13/11	0.549^b^
TBUT (s)	13.17 ± 2.26	12.37 ± 1.97	0.177^a^
Schirmer’s I test score (mm)	12.74 ± 2.56	12.04 ± 2.51	0.331^a^
OSDI score	15.24 ± 8.59	17.94 ± 7.78	0.287^a^
LLT (nm)	87.78 ± 13.55	79.21 ± 18.32	0.065^a^

*TBUT* tear break up time, *OSDI* ocular surface disease index, *LLT* lipid layer thickness.

^a^Results of continuous parameters between the two groups from an independent *t*-test.

^b^Results of non-continuous parameters between the two groups from chi-square analysis.

**Figure 2:**
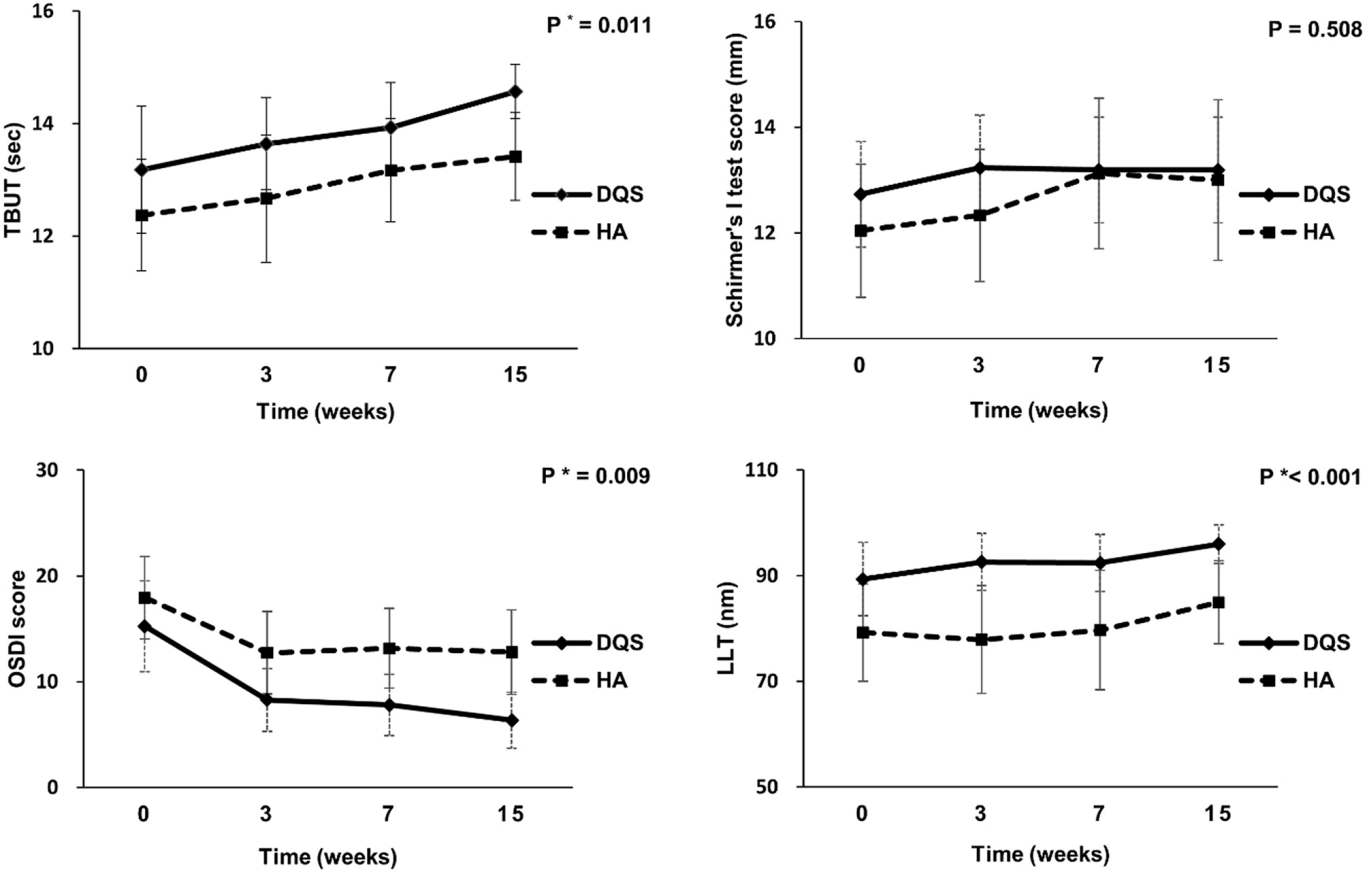
Changes in tear break up time (TBUT), Schirmer’s I test score, ocular surface disease index (OSDI) score, and lipid layer thickness (LLT). The TBUT, OSDI, and LLT during the follow-up after cataract surgery were significantly different between the subjects treated with diquafosol and hyaluronic acid (*p* = 0.011, 0.009, and < 0.001 by repeated measures ANOVA, respectively).

As mentioned in Table 2, the one-way ANOVA test revealed the significant improvement in TBUT, OSDI score, and LLT in the DQS group during the follow-up period (*p* = 0.023, < 0.001, and 0.010, respectively). Post-hoc Tukey analysis performed for each variable showed that both TBUT and LLT at postoperative 15 weeks, but not at 3 and 7 weeks, in the DQS group were significantly different from those at preoperative visit (*p* = 0.016 and 0.005, respectively, by one-way ANOVA with post-hoc Tukey test). In addition, all OSDI scores at postoperative 3, 7, and 15 weeks in the DQS group significantly varied from those reported at the preoperative visit (*p* = 0.001, 0.002, 0.001, and < 0.001, respectively, by one-way ANOVA with post-hoc Tukey test). However, all clinical parameters of the HA group showed no significant difference between the preoperative and postoperative visits. Table 2 and Fig. 2 show that the changes in TBUT, OSDI score, and LLT, but not Schirmer’s I test score, from preoperative time point to 15 weeks after cataract surgery were significantly different between the DQS group and HA group (*p* = 0.011, 0.009, < 0.001, and 0.508, respectively, by repeated measures ANOVA).

**Table 2:** Changes in TBUT, Schirmer’s I test score, ocular surface disease index (OSDI), and lipid layer thickness (LLT) in the DQS group and the HA group.

	Preoperative	Postoperative 3 weeks	Postoperative 7 weeks	Postoperative 15 weeks	*p*^a^	*p*^b^ pre vs 3 weeks	*p*^c^ pre vs 7 weeks	*p*^d^ pre vs 15 weeks	*p* value*
**TBUT**									
DQS	13.17 ± 2.26	13.64 ± 1.68	13.93 ± 1.61	14.57 ± 0.96	**0.023**	0.734	0.351	**0.014**	**0.011**
HA	12.37 ± 1.97	12.67 ± 2.25	13.16 ± 1.83	13.41 ± 1.56	0.235	0.953	0.486	0.245	
**Schirmer’s I test score**									
DQS	12.74 ± 2.56	13.23 ± 2.93	13.22 ± 3.11	13.44 ± 3.12	0.908	0.931	0.931	0.931	0.508
HA	12.04 ± 2.51	12.33 ± 2.49	13.12 ± 2.84	13.00 ± 3.03	0.457	0.983	0.519	0.619	
**OSDI**									
DQS	15.24 ± 8.59	8.28 ± 5.92	7.80 ± 5.81	6.36 ± 5.27	** < 0.001**	**0.002**	**0.001**	** < 0.001**	**0.009**
HA	17.94 ± 7.78	12.74 ± 7.77	13.16 ± 7.54	12.79 ± 7.97	0.119	0.174	0.237	0.181	
**LLT**									
DQS	87.78 ± 13.55	92.57 ± 10.79	92.42 ± 10.80	95.96 ± 7.22	**0.010**	0.321	0.348	**0.004**	** < 0.001**
HA	79.21 ± 18.33	77.87 ± 20.41	79.67 ± 22.59	84.96 ± 15.74	0.606	0.995	1.000	0.735	

Statistically significant *p* values are marked in bold (*p* < 0.05).

*TBUT* tear break up time, *OSDI* ocular surface disease index, *LLT* lipid layer thickness.

*p value < 0.05 by repeated-measures analysis of variance.

^a^*p* value < 0.05 one-way analysis followed by post-hoc Tukey analysis.

^b^Between preoperative visit and postoperative 3 weeks.

^c^Between preoperative visit and postoperative 7 weeks.

^d^Between preoperative visit and postoperative 15 weeks.

Correlation coefficients were calculated to evaluate the effects of preoperative clinical measurements on TBUT, Schirmer’s I test score, OSDI score, and LLT at postoperative 15 weeks (Table 3). TBUT at postoperative 15 weeks positively correlated with preoperative TBUT and DQS treatment (*p* = 0.012 and p = 0.002, respectively), while Schirmer’s I test score positively correlated with preoperative Schirmer’s I test score (*p* < 0.001). The OSDI score negatively correlated with age and DQS treatment and positively correlated with preoperative OSDI score (*p* = 0.007, 0.003, and 0.010, respectively). LLT showed a positive correlation with age and preoperative LLT (*p* = 0.002 and < 0.001, respectively).

**Table 3:** Pearson’s correlation coefficients between various demographic and preoperative clinical parameters and clinical measurements at postoperative 15 weeks.

Parameters	At postoperative 15 weeks			
	TBUT	Schirmer’s I test score	OSDI score	LLT
Age (year)	0.253	0.060	− 0.401**	0.189
Gender (reference: male)	− 0.089	− 0.032	0.070	− 0.002
Group (reference: HA group)	0.420**	0.037	− 0.444**	0.425**
Preoperative TBUT	0.356*	0.013	− 0.143	0.264
Preoperative Schirmer’s I test score	0.263	0.624**	− 0.145	0.271
Preoperative OSDI score	0.140	0.182	0.387*	0.062
Preoperative LLT	0.225	0.030	− 0.176	0.479**

*HA* hyaluronic acid, *TBUT* tear break up time, *OSDI* ocular surface disease index, *LLT* lipid layer thickness.

**p < 0.01 and *p < 0.05 by Pearson’s correlation analysis.

Multivariate linear regression analysis was performed to examine the influence of independent preoperative parameters on the TBUT, Schirmer’s I test score, OSDI score, and LLT at postoperative 15 weeks (Table 4). The 3% DQS treatment and preoperative TBUT were significant independent parameters for postoperative TBUT (R^2^ = 0.261, *p* = 0.016 and 0.043, respectively). Further, preoperative Schirmer’s I test score was a significant independent parameter for postoperative Schirmer’s I test score (R^2^ = 0.341, *p* < 0.001), while younger age, 3% DQS treatment, and preoperative OSDI score were significant independent parameters for postoperative OSDI score (R^2^ = 0.423, *p* = 0.005, 0.005, and 0.015, respectively). In addition, 3% DQS treatment and preoperative LLT served as significant independent parameters for postoperative LLT (R^2^ = 0.362, *p* = 0.022 and 0.040, respectively) at 15 weeks after cataract surgery.

**Table 4:** Multiple regression analysis to determine the influence of independent preoperative parameters on each clinical measurement at postoperative 15 weeks.

Parameters	Postoperative 15 weeks							
	TBUT		Schirmer’s I test score		OSDI score		LLT	
	ß (95% CI)	*p* value*	ß (95% CI)	*p* value*	ß (95% CI)	*p* value*	ß (95% CI)	*p* value*
Age (year)					− 0.375 (− 0.633, − 0.117)	0.005		
Gender (reference: male)								
Group (reference: HA group)	0.934 (0.184, 1.684)	0.016			− 5.229 (− 8.801, − 1.657)	0.005	8.517 (1.273, 15.760)	0.022
Preoperative TBUT	0.154 (0.005, 0.304)	0.043						
Preoperative Schirmer’s I test score			0.665 (0.377, 0.953)	< 0.001				
Preoperative OSDI score					0.272 (0.057, 0.488)	0.015		
Preoperative LLT							0.215 (0.003, 0.433)	0.040

*HA* hyaluronic acid, *TBUT* tear break up time, *OSDI* ocular surface disease index, *LLT* lipid layer thickness.

*Results of the backward method in the multivariate analysis.

## Discussion

The aim of this prospective, randomised, clinical trial was to compare the effectiveness of 3% DQS with that of 0.1% HA on the ocular surface following cataract surgery. We found that the changes in TBUT, OSDI score, and LLT during the 15 weeks of follow-up after cataract surgery were significantly different between the DQS group and HA group. At 15 weeks post-surgery, TBUT, OSDI score, and LLT were significantly different from the baseline outcomes in the DQS group. Postoperative treatment with 3% DQS rather than 0.1% HA showed a significant positive correlation with TBUT, OSDI score, and LLT at 15 weeks, while preoperative baseline values of each parameter significantly affected the postoperative outcomes at 15 weeks.

Corneal nerve transection, prolonged microscope light exposure, use of aspirating speculum, and heat from phacoemulsification devices could be possible risk factors for postoperative DES. The irritated surface tends to gather chemical mediators such as free radicals in response to inflammation, thereby damaging the ocular surface structure and consequently inducing evaporative DES. Another approach suggests that postoperative goblet cell loss and meibomian gland dysfunction (MGD) after cataract surgery cause evaporative DES, owing to the decrease in the production of mucin and other lipid components of the tear^12,13^. DQS may have a positive effect on improving the ocular surface following cataract surgery^14–19^, as several previous studies have suggested the following hypothesis on the mechanism of action of DQS. Animal studies indicated that DQS acts as an agonist of the P2Y2 purinergic receptor, which is profound in the conjunctiva, stimulating fluid and chloride ion secretion. In addition, it was observed that DQS increases the expression of P2Y2 receptors on conjunctival epithelial cells, increasing the production of mucin and other aqueous tear components^20^. Furthermore, a recent study suggested that DQS helps in the wound healing process of the damaged cornea by stimulating epidermal growth factor receptor/extracellular-signal-regulated kinase (EGFR/ERK) signalling-mediated cell proliferation^21^.

Many studies demonstrated the improvement in TBUT and ocular staining score after treatment with 3% DQS in DES patients^10,14^, and several studies have examined the benefits of 3% DQS on postoperative DES in cataract surgery. Lee et al. observed that postoperative TBUT and corneal staining score were significantly higher in DQS group than in HA group^18^. Park et al. concluded that the postoperative use of 3% DQS could significantly improve TBUT, corneal fluorescein stain score, and conjunctival lissamine stain score as compared to HA^22^. Although some other studies have examined the effect of DQS on DES after cataract surgery^15,17,19,23^, the included subjects had already had DES. The present study recruited patients with no ocular surface diseases, including DES, and revealed that the treatment of healthy eyes with 3% DQS could also effectively improve TBUT after cataract operation. We did not compare corneal or conjunctival staining scores because all subjects had neither DES nor ocular surface disease.

The Schirmer’s I test score showed unremarkable results at all follow-up time points. The Schirmer’s I test is known to have variable results, poor reproducibility, and low sensitivity for detecting dry eye; therefore, the result may not accurately reflect the changes in the tear film. Regarding the OSDI score, the present study showed significant improvement in the OSDI score at all three follow-up time points from baseline outcome in the DQS group. As reported before^22^, 3% DQS seems to alleviate subjective DES symptoms better than HA, implying that it significantly reduces the postoperative ocular surface symptoms. Although no significant changes were observed during the follow-up periods in the HA group, the mean OSDI score at all postoperative visits decreased. Thus, the subjective ocular surface symptoms of the HA group also improved. Furthermore, the small number of total subjects and the use of generic merchandise may have influenced the OSDI scores of HA group.

The lipid layer lies on outermost surface of the tear film and is known to stabilise the tear film^24^. It was reported that in the absence of the lipid layer, the evaporation rate of the tear was fourth-fold^25^. Thus, the LLT is considered as one of the useful parameters to evaluate the evaporative type of DES. However, few studies have measured LLT in DES patients. Kang et al. first reported the positive effect of DQS on LLT in DES patients using a tear interferometer and suggested a correlation between DQS and secretion of lipid components in tear film^16^. Yokoi et al. reported that instillation of DQS in a healthy patient’s eye could elevate the curvature and height of the tear meniscus, suggestive of a stimulating effect of DQS on tear secretion^9^. Our study focused on postoperative changes in LLT and found that 3% DQS was superior to 0.1% HA in improving LLT and that the change from baseline was significant at postoperative 15 weeks.

To our knowledge, the present study is the first to evaluate the quantitative change in LLT and confirm the determinant factors of postoperative parameters in healthy patients who used 3% DQS after cataract surgery. We found that better TBUT, Schirmer’s I test score, OSDI score, and LLT at preoperative visit could indicate improved postoperative TBUT, Schirmer’s I test score, OSDI score, and LLT, respectively, and that 3% DQS treatment following cataract surgery was a significant determinant factor of TBUT, OSDI score, and LLT at postoperative 15 weeks. As present study indicates that preoperative ocular surface significantly affected the postoperative ocular surface status, we highlight the necessity of managing ocular surface diseases before cataract surgery. In addition, the postoperative use of 3% DQS may be more advantageous than HA in managing the ocular surface after cataract surgery.

Although this study has the advantages of a double-masked, randomised, prospective assessment, it also has a few limitations. First, the study was conducted on a relatively small number of subjects and, the total follow-up period of 15 weeks is relatively short, although ocular surface changes due to phacoemulsification surgery mostly resolve within several months^26^. Second, it could be a potential limitation that this comparative study did not include a control group with no postoperative treatment for DES such as HA or DQS. However, since there have been several previous studies that compared HA with placebo and DQS with placebo in which the results proved the significant efficacy of each eye solution in DES, the present study did not include a control group which received placebo^27,28^. Finally, postoperative antibiotics and anti-inflammatory agents such as levofloxacin and fluorometholone, could have positively influenced ocular surface status. However those medication was equally used in same way in both groups, which implies that the choice between the use of either DQS or HA might have made the difference between the groups.

In conclusion, the present study demonstrates that the postoperative TBUT and LLT significantly increased in the DQS group compared with the HA group. Also, they showed significant positive correlation with postoperative use of 3% DQS, preoperative TBUT and LLT. Therefore, it may be more effective to manage postoperative ocular surface with 3% DQS than with the conventionally used 0.1% HA following cataract surgery.

## Data Availability

Data are available upon reasonable request. Data supporting our research are available upon a reasonable request.
